# Developmental and Digestive Flexibilities in the Midgut of a Polyphagous Pest, the Cotton Bollworm, *Helicoverpa armigera*

**DOI:** 10.1673/031.012.4201

**Published:** 2012-03-24

**Authors:** P.J. Sarate, V.A. Tamhane, H.M. Kotkar, N. Ratnakaran, N. Susan, V.S. Gupta, A.P. Giri

**Affiliations:** Plant Molecular Biology Unit, Division of Biochemical Sciences, National Chemical Laboratory, Dr. Homi Bhabha Road, Pune 411 008 (M.S.), India; # these authors contributed equally to the work

**Keywords:** amylases, larval performance, lipases, proteases

## Abstract

Developmental patterns and survival of the cotton bollworm, *Helicoverpa armigera* Hübner (Lepidoptera: Noctuidae), a polyphagous insect pest, have been studied with reference to the effect of diet on major gut digestive enzymes (amylases, proteases, and lipases). Significant correlations between nutritional quality of the diet and larval and pupal mass were observed when *H. armigera* larvae were fed on various host plants *viz*. legumes (chickpea and pigeonpea), vegetables (tomato and okra), flowers (rose and marigold), and cereals (sorghum and maize). Larvae fed on diets rich in proteins and/or carbohydrates (pigeonpea, chickpea, maize, and sorghum) showed higher larval mass and developed more rapidly than larvae fed on diets with low protein and carbohydrate content (rose, marigold, okra, and tomato). Low calorific value diets like rose and marigold resulted in higher mortality (25–35%) of *H. armigera*. Even with highly varying development efficiency and larval/pupal survival rates, *H. armigera* populations feeding on different diets completed their life cycles. Digestive enzymes of *H. armigera* displayed variable expression levels and were found to be regulated on the basis of macromolecular composition of the diet. Post—ingestive adaptations operating at the gut level, in the form of controlled release of digestive enzymes, might be a key factor contributing to the physiological plasticity in *H. armigera*.

## Introduction

The cotton bollworm, *Helicoverpa armigera* Hübner (Lepidoptera: Noctuidae) is one of the most devastating insect pests worldwide, infesting about 300 plant species. It is a rapacious feeder that causes significant reductions in yield of economically important crops like chickpea, maize, sorghum, sunflower, cotton, tobacco, soybean, pulses, safflower, rapeseed, and groundnuts (Fitt 1989; Arora et al. 2005; Rajapakse and Walter 2007). Host—pest interactions have gained importance in the scenario of increased pest resistance and adaptation. Co—evolution of host resistance and pest adaptation has led to the development of unique strategies for plants and pests to overcome each other's mechanism(s) for survival. Generalist feeders like *H. armigera* oviposit on a wide range of nutritionally different hosts and consequently cause major yield losses in crop plants (Reed and Pawar 1982; Zalucki et al. 1986; Fitt 1989; Babic et al. 2008). In nature, early instars of *H. armigera* usually feed on leaves low in nutrients, and during further growth they encounter reproductive structures that are nutrient rich. *Helicoverpa armigera* is able to survive in highly adverse conditions due to features such as polyphagy, high mobility, high fecundity, and facultative diapauses (Fitt 1989). The ability of insects to survive on diverse host plants is an adaptive mechanism for their survival in the ecosystem (Subramaniam and Mohankumar 2006). Polyphagy requires physiological mechanisms to confront the varying chemical complexities posed by different host plants. Behavioral features such as selective foraging/feeding and physiological processes like digestion, absorption and allocation are responsible for insect nutrition (Raubenheimer and Simpson 1998). The relative as well as the total amounts of protein, carbohydrate, and lipid in the diet directly influence insect growth and development. Insects tune themselves to maximize benefits and minimize costs by making proper nutrition decisions, which in turn reflect on growth and reproduction (Babic et al. 2008). Insects have mechanisms to cope with digesting protein—rich plant reproductive structures, carbohydrate—rich leaves, and even diverse unbalanced diets (Simpson and Raubenheimer 1993).

The gut is the principal site for secretion of digestive enzymes, digestion of food and absorption of nutrients (Pauchet et al. 2007). Macromolecular components present in the foods are catalyzed by three major digestive enzymes in the gut *viz*. amylases: EC 3.2.1.1, proteases: EC 3.4.21, and lipases: EC 3.1.1.3. Amylases catalyze the hydrolysis of α-D-(1, 4)-glucan linkage in starch, glycogen, and other carbohydrates, which serve as energy sources for insect larvae (Johnston et al. 1991; Purcell et al. 1992; Pereira et al. 1999; Franco et al. 2000). Serine proteinases, such as trypsin and chymotrypsin, are predominant in the gut of lepidopteran insects. Gut proteases further release amino acids from peptides produced by endopeptidases (Terra and Ferreira 1994; Terra and Cristofoletti 1996). Our earlier studies indicated that changes in the gut proteases and amylases are related to host diet content (Patankar et al. 2001; Kotkar et al. 2009). Other important but less studied enzymes from lepidopteran insects are lipases, which catalyze the hydrolysis of triacylglycerol (TAG). Lipases secreted into the midgut lumen of insects break down a variety of dietary lipids, such as triacylglycerol and phospholipids, into fatty acids (Weintraub and Tietz 1973). Among insects, most studies have focused on the roles of lipases in the fat body as compared to the gut digestive system. Grillo et al. (2007) described the role of TAG lipase in lipid digestion in the *Rhodnius prolixus* midgut. The products of digestion are absorbed by the midgut epithelium and then used to synthesize complex lipids, such as TAGs, diacylglycerols, and phospholipids. In *Bombyx mori*, lipases are not only digestive proteins but also act as antivirals against the occlusion derived nucleopolyhedrovirus (Ponnuvel et al. 2003). Home and Haritos (2008) recently reported a neutral lipase gene cluster in *Drosophila* and proposed that the lipase cluster has undergone dynamic evolutionary changes to maximize absorption of lipid nutrients from the diet. Effects of classical insect hormones on the expression profiles of a lipase gene from *H. armigera* have been studied (Sui et al. 2008).

Our earlier results provided insights for correlating growth and development of *H. armigera* on nutritionally varied host plants with major digestive enzymes. We hypothesize macromolecules (protein, carbohydrates, and lipids) in the host plants dramatically influence *H. armigera* growth and development mediated through qualitative and quantitative regulation of digestive enzymes. The hypothesis was tested by tracking the growth and development of *H. armigera* on eight nutritionally diverse natural diets. Activity assays of digestive enzymes (proteases, amylases, and lipases) of the larval stage were then correlated both with the nutritional value of the diet and the developmental response on the same diet. Results indicate that *H. armigera* has a highly developed, enzyme regulation mechanism favoring its survival and polyphagy. Such studies that explore the polyphagous insect's perspective when exposed to varied diets will help in better pest control strategy design.

## Materials and Methods

### Insect culture and feeding assays

*Helicoverpa armigera* larvae were collected from chickpea fields of Mahatma Phule Krishi Vidyapeeth (MPKV), Rahuri, India, and maintained on a chickpea—flour based artificial diet under laboratory conditions of 28 ± 2 °C and 75% RH. Artificial diet composed of (A) 50 g chickpea flour, 12 g yeast extract, 5 g wheat germ, 3.5 g casein, 0.5 g sorbic acid, and 1 g methyl paraben in 150 mL distilled water; (B) 0.35 g choline chloride, 0.02 g streptomycin sulphate, 2 g ascorbic acid, 0.15 g cholesterol, Becadexamin multivitamin multi—mineral capsule (GlaxoSmithKline Pharmaceuticals Limited, www.gsk.com), 200 mg Vitamin E, 1 mL formaldehyde, 0.3 g bavistin, 30 mL distilled water; and (C) 6.5 g agar in 180 mL distilled water. ‘A’ and ‘B’ were mixed together and molten agar ‘C’ was added (Nagarkatti and Prakash 1974). Neonates from the following generation were fed on artificial diet up to the first instar and then transferred onto eight natural host plant tissues, namely whole pods of the legumes chickpea (*Cicer arietinum* L. var. Digvijay) and pigeonpea (*Cajanus cajan* L. var. Vipula), fresh cut vegetables green unripe tomato (*Lycopersicon esculentum* L. var. Pusa Ruby) and okra (*Abelmoschus esculentus* S. var. Parbhani Kranti), whole flowers rose (*Rosa* sp. var. Ruby Red) and marigold (*Tagetus erecta* var. African marigold), and cereals in the form of portions of sorghum cobs (*Sorghum bicolor* var. Hybrid Madhura) and kernels of maize (Zea *mays* L. var. Saccharata). Neonates were also fed on the artificial diet for comparison. Samples of the plant tissues from each diet were stored at -80 °C and 4 °C for biochemical analysis and insect feeding, respectively. One hundred neonates were transferred on each diet and supplied with fresh plant tissue daily. The insects were maintained at 28 ± 2 °C and 75% RH, and were weighed every other day starting from the second instar up to the pupal stage. Mid—fourth instar larvae (30) feeding on each of the above mentioned diets were anaesthetized using chloroform. Insects were then dissected ventrally by sharp surgical blade and guts were removed by using pointed forceps, followed by immediate snap freezing in liquid nitrogen. They were stored at -80 °C for further use.

The following observations were recorded during the feeding assays: (i) mass gained by the larvae, (ii) larval period (the number of days required for a neonate larva to metamorphose into a pupa), (iii) percent mortality during first to third, fourth to fifth, sixth instar larvae, and the pupal stage, (iv) pupal mass, (v) pupation efficiency/delayed (prolonged) pupation by recording the number of larvae pupating during days 1–4 and days 5–10, with day 1 of pupation considered as the day when the first larva metamorphosed into pupa.

### Extraction of gut proteins

The gut tissue collected was homogenized in liquid N_2_ with a mortar and pestle. Then, 100 mg of gut tissue was extracted in 300 µL of 0.02 M sodium—phosphate buffer (pH 6.8) containing 10 mM NaCl for two hours at 4 °C. This mixture was then centrifuged at 13,000 × g for 30 min at 4 °C. The supernatant was collected, stored at -20 °C, and used as a source of gut enzymes for various biochemical assays. Similarly, extracts were prepared from samples of the plant tissues mentioned in the above ‘Insect culture and feeding assays’ section to estimate endogenous enzyme activities.

### Estimation of carbohydrates, proteins, and lipids

Total carbohydrate content of the plant tissues used for feeding assays was estimated by the phenol sulfuric acid method (Dubois et al. 1956) using glucose (HiMedia Laboratories, www.himedialabs.com) as a standard. Soluble protein content was determined by Bradford's method (Bradford 1976) using bovine serum albumin as a standard (Kotkar et al. 2009).

Lipids were extracted from plant tissue samples (25 mg each) using 7 mL chloroform: methanol (2:1 v/v). Following the addition of NaCl (0.9%; 1 mL), the samples were centrifuged for 10 min at 3000 × g. The aqueous phase was discarded, while the pellet was dried at 45 °C using a CentriVap Concentrator (Labconco, www.labconco.com) and dissolved in 1 mL chloroform. Lipid content was measured from this extract using the sulpho—phospho—vanillin method (Zöllner and Kirsch 1962). Concentrated H_2_SO_4_ (200 µL) was added to the extract (100 µL) and incubated at 100 °C for 10 min in a water bath. After cooling to room temperature, 2.5 mL of H_3_PO_4_-vanillin reagent (20 mL of 0.6% (w/v) vanillin solution + 80 mL of 85% H_3_PO_4_) was added. The extract was vortexed and incubated for one hour at room temperature. Absorbance was measured at 528 nm using cholesterol (Super Religare Laboratories Ltd., www.srl.in) as a standard.

Calorific values (kcal) of host plant tissues fed to *H. armigera* larvae were obtained from www.nutritiondata.com. Rose and marigold kcal values/100 g tissue were calculated using values 4 kcal/g for protein/carbohydrate and 9 kcal/g for fat www.essortment.com.

### Amylase, protease, proteinase, and lipase assay

Amylase activity in the gut of *H. armigera* larvae fed on different plant tissues was analyzed by the dinitrosalycylic acid (DNSA; Sigma-Aldrich, www.sigma-aldrich.com) method (Bernfeld 1955) as described by Kotkar et al. (2009). One amylase unit was defined as the amount of enzyme required to release 1 µM maltose/min at 37 °C under the given assay conditions.

Azocasein (Brock et al. 1982) and trypsin assays of gut homogenates and plant tissue extracts were carried out as described by Tamhane et al. (2005). One protease/proteinase unit was defined as the amount of enzyme in the assay that causes an increase in absorbance by one OD under the given assay conditions.

Lipase activity from gut homogenates and plant tissue extracts was estimated using the p-nitrophenyl palmitate (pNPP; Sigma-Aldrich) assay (Winkler and Stuckmann 1979). The substrate was comprised of solution A and solution B. Solution A contained 0.1 g gum arabica and 0.4 mL Triton X-100 (Sigma-Aldrich) dissolved in 90 mL of distilled water. Solution B contained 30 mg pNPP dissolved in 10 mL isopropanol. The substrate solution was prepared by adding 9.5 mL of solution A to 0.5 mL of solution B drop—wise with constant stirring to obtain an emulsion that was stable for two hours. The assay mixture, containing 0.9 mL of the substrate, 0.05 mL of buffer (0.02 M sodium—phosphate buffer (pH 6.8) containing 10 mM NaCl and 0.05 mL of gut extract), was incubated at 37 °C for 30 min using a thermomixer (Eppendorf, www.eppendorf.com). Enzyme activity was stopped by adding 2 mL of 1% sodium carbonate (Na_2_CO_3_). Absorbance of the samples was measured at 410 nm against a substrate—free blank.

All the assays were performed in duplicate and repeated three times. One unit of lipase activity was defined as the amount of enzyme that causes an increase of one OD under the given assay conditions.

### Statistical analysis

Significant differences between diet treatments were determined using single factor ANOVA with replication in conjunction with Tukey's post hoc Honestly Significant Difference (HSD) test. Data was considered to be significantly different within the treatments if the *F*—value obtained was higher than the critical *F*—value at a probability level of 0.01. Critical differences (CD) between HSD values were calculated by subtracting subsequent values of the averages and comparing with the calculated CD at *p* < 0.05 and *p* < 0.01 (Kotkar et al. 2009). Small letters were used to indicate statistically different groups of treatments in the Tukey's post hoc HSD.

## Results

### Total carbohydrate, soluble proteins, total lipid content, and calorific value of diets

Plant parts used for feeding assays of *H. armigera* were analyzed for total carbohydrate, soluble protein, and lipid content (Table 1). Maize had a high calorific value, with significantly higher carbohydrate content (*p* < 0.01) than all the other diets. The pigeonpea diet provided significantly higher soluble proteins (*p* < 0.01) when compared to all the other diets. Chickpea, sorghum, and tomato diets provided significantly less lipids than all the other diets (*p* < 0.05). The artificial diet contained high levels of carbohydrates and lipids, with comparably lower soluble protein levels as compared to the natural diets with high protein content (pigeonpea, chickpea, maize, and sorghum). Calorific values per 100 g of host plant tissue fed to *H. armigera* were as follows: chickpea (415 kcal), pigeonpea (355 kcal), okra (32 kcal), tomato (25 kcal), rose (39 kcal), marigold (49 kcal), sorghum (368 kcal), maize (495 kcal), and artificial diet (ND) (Table 1).

### Differences in *H. armigera* growth pattern during feeding on various diets

**Figure 1.**
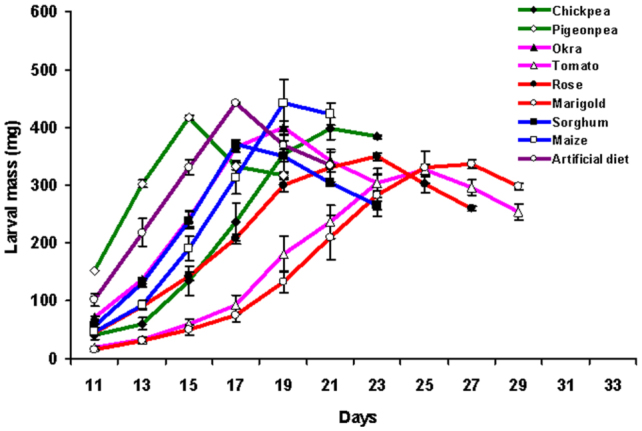
Growth pattern of *Helicoverpa armigera* larvae. Larvae fed on natural and artificial diets were analyzed for growth and development. Larval mass was recorded on every alternate day starting from the 11^th^ day after emergence from eggs. The number of days required to complete the larval stage was recorded. Error bars represent mean ± SD of three independent sets of 17 larvae on each diet. High quality figures are available online.

**Supplementary Figure 1.**
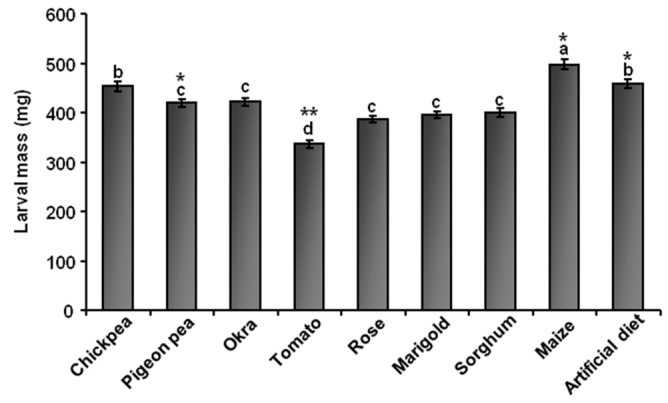
Mass analysis of *Helicoverpa armigera* larvae. Maximum larval mass of *Helicoverpa armigera* gained during feeding on natural and artificial diets. Single factor ANOVA followed by Tukey's post hoc HSD suggested significant difference between the data at *p* < 0.01 (indicated as ^**^). Similar letters represent a group with statistically insignificant difference. Error bars represent mean ± SD of three independent sets of 17 larvae on each diet. High quality figures are available online.

Larvae fed on artificial and natural diets showed unique patterns of growth (Figure 1). Larvae fed on diets rich in proteins and/or carbohydrates (legumes and cereals) showed higher larval mass and developed more rapidly than larvae fed on diets with low protein and carbohydrate content (flowers and vegetables). Pigeonpea—fed larvae completed the larval stadium in the shortest time (19 days), while larvae fed on tomato and marigold completed their larval cycle (approximately 29 days) with maximum days. Significant differences (*p* < 0.01 and *p* < 0.05) in maximum larval mass were observed between the nine diets (Supplementary Figure 1). Larvae fed on maize, the diet with the highest calorific value, gained the most mass (497.1 mg), followed by larvae fed on the artificial diet (459.3 mg) and chickpea (453.0 mg). Larvae fed on tomato, the diet with the lowest calorific value, had the lowest larval mass (336.2 mg).

**Table 1.**
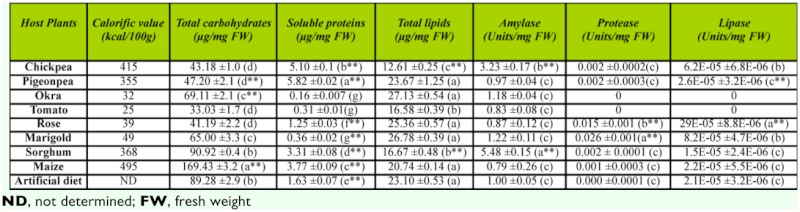
Analysis of host plant tissue for macromolecular content and endogenous enzyme activities. Shows calorific values, total carbohydrate, soluble protein, total lipid content (represented as µg/mg FW) and amylases, proteases and lipases (represented as U/mg FW) of plant tissues used for *Helicoverpa armigera* feeding assays. Analyses of diets were done as mentioned in Materials and Methods. Results are an average of three independent experiments conducted in duplicate. Error bars represent mean ± SD. Single factor ANOVA followed by Tukey's post hoc HSD suggested significant difference between the data at *p* < 0.01 (indicated as ^**^). Similar letters represent a group with statistically insignificant difference.

### Variation in *H. armigera* pupal periods and pupal weights

**Figure 2.**
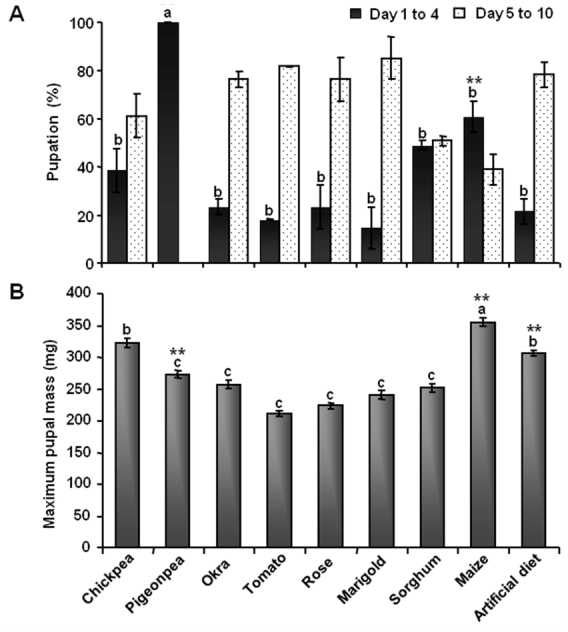
*Helicoverpa armigera* pupation analysis. (A) Percent pupation of *Helicoverpa armigera* fed on natural and artificial diets during days 1–4 and days 5–10. (B) Maximum pupal mass. Single factor ANOVA followed by Tukey's post hoc HSD suggested significant a difference between the data at *p* < 0.01 (indicated as ^**^). Similar letters represent a group with statistically insignificant difference. Error bars represent mean ± SD of three independent sets of 17 pupae on each diet. High quality figures are available online.

Significant differences between diets were observed for both pupation efficiency (Figure 2A) and pupal mass (Figure 2B). Almost 99% of pigeonpea—fed larvae attained the pupal stage in the first four days of the pupation window. Sixty percent of maize—fed larvae also reached the pupal stage within four days. For all other diets, there was no significant difference in the pupation efficiency (p < 0.01). Pupal mass followed the same trend as larval mass, mentioned above, with pupae of larvae fed on maize having the highest pupal mass (355.6 mg) followed by chickpea—fed (322.6 mg) and artificial diet—fed larvae (305.9 mg). Pupal mass was the lowest for tomato—fed larvae (211.6 mg).

### Mortality rate of *H. armigera* larvae fed on various diets

**Figure 3.**
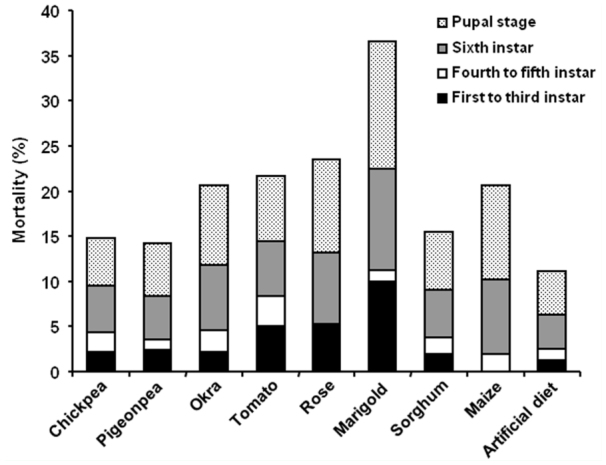
Mortality in *Helicoverpa armigera* feeding on different diets. Larvae were allowed to feed on natural and artificial diets. Mortality (%) during first to third instar, fourth to fifth instar, sixth instar, and the pupal stage when fed on various diets was ascertained. Results are from three independent sets of 17 larvae feeding on each diet. High quality figures are available online.

Diets with low protein, carbohydrate and lipid content resulted in higher mortality of *H. armigera* (Figure 3). Marigold—fed larvae had the highest mortality (36%). Mortality was the lowest in chickpea— and pigeonpea—fed larvae (14%), while artificial diet—fed larvae also showed the lowest mortality (12%). During the early instar stages (first to third), the highest mortality (10%) was observed with marigold—fed larvae. Early instar mortality was negligible in larvae fed on the artificial diet and in maize—fed larvae. During the fourth and fifth instar, mortality on all diets was insignificant (less than 2%), while it was relatively higher during the sixth instar and the pupal stage.

**Supplementary Table 1.**
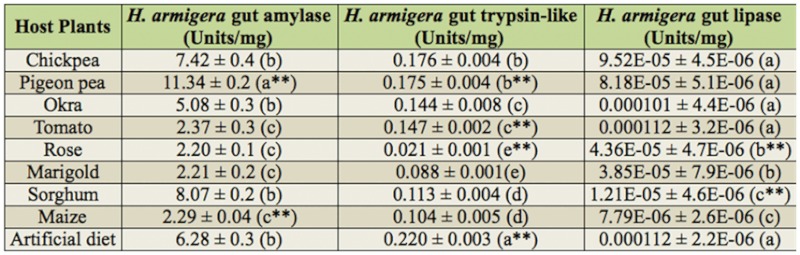
*Helicoverpa armigera* gut amylase and trypsin—like enzyme activities. Represents gut amylase and trypsin—like enzyme activities of larvae fed on natural and artificial diets. Results are an average of three independent experiments conducted in duplicate. Error bars represent mean ± SD. Single factor ANOVA and Tukey's post hoc HSD showed significant difference between the data at *p* < 0.01 (indicated by ^**^). Similar letters represent a group with statistically insignificant difference.

### Gut digestive enzymes of *H. armigera* larvae

Statistical analysis showed significant differences between gut amylase, protease, trypsin—like, and lipase activities of larvae fed on different diets at *p* < 0.01 and *p* < 0.05. Amylase activity was significantly high (*p* < 0.01) in gut extract of fourth instar larvae fed on pigeonpea (11.3 U/mg gut tissue) and was low in rose— and marigold—fed *H. armigera* (2.2 U/mg gut tissue) (Supplementary Table 1). Of the high carbohydrate diets (maize, sorghum, and the artificial diet), maize—fed larvae possessed significantly lower amylase activity (2.3 U/mg gut tissue) than sorghum—fed larvae (8.0 U/mg gut tissue) and those fed on the artificial diet (6.3 U/mg gut tissue). Endogenous amylase activity of all host plant tissues used in the feeding assays was lower (0.8 to 1.2 U/mg plant tissue) than the activity levels measured in the gut extracts (2.2 to 11.3 U/mg gut tissue), except for the sorghum and chickpea tissue (Table 1).

**Figure 4.**
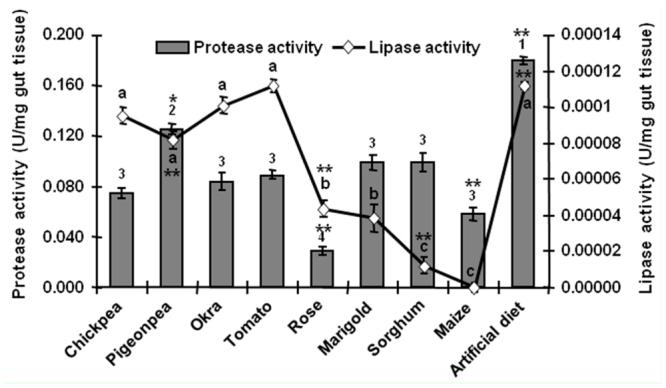
Gut protease and lipase activity of *Helicoverpa armigera* fed on various diets. Gut extracts of *Helicoverpa armigera* larvae fed on natural and artificial diets were analyzed for protease and lipase activities. Statistical analysis using single factor ANOVA and Tukey's post hoc HSD showed significant a difference between the enzyme activities of gut extract of larvae fed on different diets at *p* < 0.01 (indicated by ^**^) and at *p* < 0.05 (indicated as ^*^). Results are an average of three independent experiments conducted in duplicate. Error bars represent mean ± SD. Similar letters represent a group with statistically insignificant difference in gut enzyme levels. High quality figures are available online.

Larvae fed on pigeonpea, the diet with the highest protein content, possessed significantly higher (*p* < 0.01) gut protease (Figure 4) and trypsin—like activity (Supplementary Table 1) (0.12 U/mg and 0.17 U/mg gut tissue, respectively) than all the other natural diets. Among the cereal diets, sorghum—fed larvae showed higher total gut protease activity (0.1 U/mg gut tissue) than maize—fed larvae (0.05 U/mg gut tissue), yet larvae from both diets showed similar trypsin—like activity (0.1 U/mg gut tissue). The lowest levels of protease and trypsin—like activity were observed in larvae fed on diets that provided the lowest amounts of soluble protein (flowers and vegetables). Endogenous total protease activity of marigold (0.026 U/mg plant tissue) and rose (0.015 U/mg plant tissue) used for the feeding assays were significantly high (*p* < 0.01), whereas it was either very low or undetectable in all the other diets (Table 1).

Comparison of lipase activity in the gut extracts of larvae fed on diets that provided the lowest lipid content (chickpea, tomato, sorghum) revealed significantly high lipase activity in tomato— and chickpea—fed larvae (11.2 × 10^-5^ U/mg and 9.52 × 10^-5^ U/mg gut tissue, respectively), yet in contrast, low lipase activity in sorghum—fed larvae (1.2 × 10^-5^ U/mg gut tissue). Endogenous lipase activity of diets used for *H. armigera* feeding assays (Table 1) revealed significantly high levels in rose (29.9 × 10^-5^ plant tissue U/mg). Marigold and chickpea formed a second group with comparatively moderate lipase activity (8.27 × 10^-5^ U/mg and 6.21 × 10^-5^ U/mg plant tissue, respectively), while all the remaining diets showed low lipase activities.

## Discussion

Plant—insect interactions are dependent on nutritional levels of plant tissues during different growth forms of the insect and chemical and mechanical defenses of the plant (Cates 1980). Polyphagous insects feed on many host plants and enjoy a better chance of survival in nature (Ambika et al. 2005). Insect diets comprise varying proportions of organic constituents belonging to three major groups, *viz*. proteins, carbohydrates, and fats, which directly affect insect growth and reproduction. We observed that *H. armigera* successfully completes its life cycle on nutritionally diverse host plants and probably undergoes metabolic adjustments to achieve the same. In this context, we have explored the regulation of digestive enzyme activities, growth, and development of *H. armigera* fed on different diets.

### *Helicoverpa armigera* displays developmental flexibility while feeding on different hosts

When fed on different natural diets, *H. armigera* showed significant differences in terms of larval and pupal mass. Insects grew faster and attained higher larval and pupal mass when fed on pigeonpea and maize, while delayed larval growth and lower larval and pupal mass were observed in *H. armigera* feeding on vegetables and flowers. Awmack and Leather (2002) reported that lepidopteran larvae fed on nutritive and nitrogen rich diets have a high pupal mass. Larval performance was found to be largely dependent on the proportion of protein and carbohydrate present in the diets. Fefelova and Frolov (2008) have reported that maize is one of the most preferred hosts of *H. armigera*. This could be because *H. armigera* performs better on diets with balance in carbohydrate and protein content.

The efficiency of pupation was directly influenced by larval mass gained. Differing nutrient levels in various host plants have been shown to affect over—wintering in *H. armigera* pupae (Liu et al. 2009). Late larval stages serve to acquire energy reserves for the maintenance of the adult insect form (Lorenz and Gade 2009). In this study, *H. armigera* larvae fed on pigeonpea pupated early and synchronously. Pupation was delayed by 10 or more days in *H. armigera* feeding on vegetables and flowers. In support of these findings, Bouayad et al. (2008) observed latency in pupation by 20 days in *Plodia interpunctella* larvae when raised on a diet that was poor in both proteins and carbohydrates.

Polyphagous insects prefer nutritionally balanced food in terms of both macro and micronutrient constituents, but imbalanced food(s) can also satisfy their nutrient requirements (Raubenheimer and Simpson 1999). For example, rose— and marigold—fed *H. armigera* successfully completed their life cycles. These flower crops have been reported to incur economic losses due to *H. armigera* infestation. Survival rates of *H. armigera* were higher for larvae fed on chickpea, pigeonpea, and maize as a diet and lower for those fed on okra, tomato, rose, and marigold. Adjusting to deficiencies in diet is an energy demanding process and may sometimes prove to be fatal. The rate of insect mortality at a particular larval stage is indicative of failure of the insect's adaptability to a particular diet.

Considerably high mortality was recorded in very early and late stages of *H. armigera*. Due to their undeveloped mouthparts, neonates are susceptible to death if fed on diets that are difficult to chew (Zalucki et al. 1986). During their high intake stages (fourth and fifth instar), *H. armigera* larvae showed low mortality on all diets, irrespective of the nutritive value (Figure 3). Larval feeding lessens after the late fifth instar and almost stops during the sixth instar, wherein the larvae spend energy preparing for pupation. Consequences of feeding on diets imbalanced in their macromolecular content are reflected in the survival rate of late larval and pupal stages. Overall, studies indicate that larvae not only try to complete their life cycle on nutritionally poor diets but also extend their larval period to gain nutrition for better survival. Nutritionally rich diets help *H. armigera* to complete its life cycle early, promoting its multivoltine nature, and thus exhibiting developmental flexibility.

### *Helicoverpa armigera* adaptation on various hosts is mediated at the digestive enzyme level

The present study highlights that *H. armigera* has a highly developed, functional, post—ingestive nutrient balancing system responsible for its survival and polyphagy. When a homogenous population of insects is forced to feed on different diets—highly variable in terms of calorific value—they differentially regulate the release of their digestive enzymes.

The first stage at which the post—ingestive enzyme rebalancing might occur is within the gastrointestinal tract (GIT). Our studies indicated higher gut amylase activity in pigeonpea and chickpea—fed larvae as compared to those fed on maize (Kotkar et al. 2009; present study), i.e., the gut amylase levels were inversely proportional to the diet carbohydrate content. This supports earlier observations that insects release less of the enzymes for nutrients present in excess, while maintaining or boosting levels of enzymes for nutrients in deficit (Kotkar et al. 2009; Lwalaba et al. 2010). Lwalaba et al. (2010) reported that *Spodoptera frugiperda* larvae adjust the release of amylases in response to the level of carbohydrate intake. Clissold et al. (2010) used the locust system, feeding on artificial and natural diet(s), to show that compensatory responses occur within the GIT to bring about differential release of proteases and amylases for matching the nutritional state of the diet.

Sorghum—fed *H. armigera* gut extracts showed high amylase activity compared with the level found in larvae fed on maize. This difference in *H. armigera* gut amylase levels when fed on two starch—rich diets could be attributed to i) amount of free sugars and nature/complexity of starch in two diets and ii) up regulation of gut amylases due to presence of amylase inhibitors in the developing seeds of sorghum. Amylase activity in okra—fed larvae was high, even though okra has high carbohydrate content. This could be due to the gummy carbohydrates consisting of galacturonic acid, galactose, rhamnose, and glucose trapped in mucilaginous fruits of okra (Woolfe et al. 1977). The ratio of constituent amylose and amylopectin is an important feature of starch complexity and determines its digestibility (Moore et al. 2008). Development of *H. armigera* is better when it feeds on maize then when it feeds on sorghum, which inversely correlates with the gut amylase levels (less in maize—fed larvae more in sorghum fed larvae).

*Helicoverpa armigera* digestive gut proteases are complex, diverse, and flexible during larval development and upon feeding on various host plants at the qualitative and quantitative level (Patankar et al. 2001; Chougule et al. 2005; Kotkar et al. 2009). Gut protease levels were found to be similar in *H. armigera* fed on diets with varying protein content (for example, chickpea, okra, marigold, and sorghum). Proteins are essential for larval growth and egg production in the adult moth. Moreover, protein intake/assimilation occurs exclusively during the larval stages (Sorge et al. 2000). Larvae, therefore, cannot afford to allow any dietary protein to pass undigested through the gut. Thus, it is advantageous to maintain a constant level of trypsin/protease release in the event that even a small amount of protein is ingested (Lwalaba et al. 2010). It is a physiological necessity to maintain protease levels even in poor protein diets. Larvae feeding on low protein diets bear the cost to maintain the gut protease levels, as reflected by their prolonged larval/pupal stage and low mass.

Lipases play an important role in the physiology of insects, particularly at the non—larval life stages (Kristanto 2006). Several other insects also possess and express digestive lipases and show a dynamic evolutionary variation in their gene clusters, pointing towards their importance in the digestive physiology (Ponnuvel et al. 2003; Grillo et al. 2007; Home and Haritos 2008). However, in our study we relate the diet—dependent variation in the gut lipase content of *H. armigera*. We observed an interesting correlation in the gut lipase levels, host plant lipase levels, and protein and carbohydrate content of the diet. First, pigeonpea, a high protein and low carbohydrate content diet, and maize, a moderate protein and high carbohydrate content diet, both provided similar levels of total lipid. However, gut lipases were high in pigeonpea—fed larvae, while they were low in maize—fed larvae. The probable explanation for high lipases in a diet with high protein and low carbohydrates is i) lipase biosynthesis is easily achieved due to amino acids sourced from high dietary protein and ii) there is a need for dietary lipid breakdown to compensate for the low dietary carbohydrate. These results are comparable to earlier findings of Lwalaba et al. (2010), which reported low *S. frugiperda* lipase activity on diets with high carbohydrate content. Second, okra and marigold both have low protein and carbohydrate content; however, the gut lipase activity of *H. armigera* feeding on these diets was found to be contrasting. Correlating this further with diet lipases, okra had no detectable level of endogenous lipase activity, whereas marigold had significantly high endogenous lipase levels. Although the fate of food lipases in its digestive system is not completely known, results indicate that *H. armigera* uses the endogenous lipase activity of the host plant tissue to accordingly modify its gut lipase level, preventing further investment in terms of amino acids and energy for lipase synthesis.

**Figure 5.**
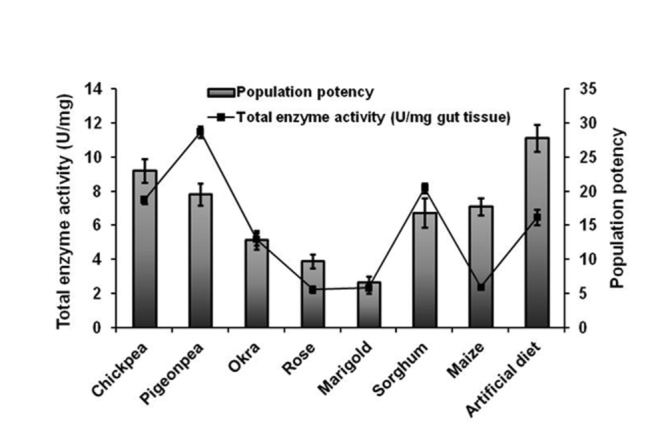
Comparison of total enzyme activity with population potency of *Helicoverpa armigera* fed on various diets. Total enzyme activity for amylase, protease and lipase of *H. armigera* larvae fed on natural and artificial diets is correlated with population potency. Population potency is the ratio of pupal mass and mortality. Error bars represent mean ± SD. High quality figures are available online.

Insect enzyme regulation mechanism(s) in *H. armigera* seem to be highly evolved. The host plant proteins, carbohydrates, and lipids influence the process of gut enzyme regulation in *H. armigera*. The host plant endogenous enzymes/enzyme inhibitors and complexity of diet composition were also found to direct the regulatory process. Expressing digestive enzymes is an energy—and protein—demanding process that might have direct implications on insect fitness, as demonstrated by larval/pupal weight and insect growth rate. In insects feeding on low calorific diets, up regulation of digestive enzyme levels and maintaining them throughout insect development is particularly resource demanding. Our studies indicated that while feeding on nutritionally poor diets, *H. armigera* maintained moderate gut protease levels reflected by significantly slower larval development and lower larval and pupal mass. Insects try to maintain the level of proteases and regulate expression of amylase and lipases with respect to their dietary substrate levels. Balancing the allocation of assimilated nutrients towards insect development and expression of digestive enzymes is a key to physiological adaptations in *H. armigera*. These mechanisms are largely responsible for the highly polyphagous nature of the pest. To explain this further, population potency (a ratio of pupal mass and mortality) was calculated. It was compared with total enzyme activity in the gut (amylases, proteases, and lipases). When population potency is high, *H. armigera* seems to invest less energy in expressing digestive enzymes (Figure 5). This strongly indicates that composition of diet directly influences the digestive plasticity in association with the development of *H. armigera*.

This study helps us in understanding the extent of digestive and developmental flexibility that *H. armigera* possesses. Any further pest control strategy developed using the gene silencing or transgenic crop approach can turn out to be sustainable when this adaptive dynamism is taken into consideration. Unraveling the molecular mechanisms controlling these adaptive changes in *H. armigera* is also crucial for providing leads that could be developed for crop plant protection.
